# Finite element analysis of archwire parameters and activation forces on the M/F ratio of vertical, L- and T-loops

**DOI:** 10.1186/s12903-020-1059-z

**Published:** 2020-03-13

**Authors:** Yongqing Cai

**Affiliations:** Department of Mechanical and Electrical Engineering, Hainan University, Present address: #58 People Avenue, Meilan District, Haikou City, Hainan Province China

**Keywords:** M/F ratio, Loop mechanics, Loop design parameter, Activation force, Finite element analysis

## Abstract

**Background:**

The ability of a loop to generate a certain moment/force ratio (M/F ratio) can achieve the desired tooth movement in orthodontics. The present study aimed to investigate the effects of elastic modulus, cross-sectional dimensions, loop configuration geometry dimensions, and activation force on the generated M/F ratio of vertical, L- and T-loops.

**Methods:**

A total of 120 three-dimensional loop models were constructed with the Solidworks 2017 software and used for simulating loop activation with the Abaqus 6.14 software. Six vertical loop variations, 9 L-loop variations, and 9 T-loop variations were evaluated. In each group, only one parameter was variable [loop height, ring radius, leg length, leg step distance, legs distance, upper length, different archwire materials (elastic modulus), cross-sectional dimension, and activation force].

**Results:**

The simulation results of the displacement and von Mises stress of each loop were investigated. The maximum displacement in the height direction was recorded to calculate the M/F ratio. The quantitative change trends in the generated M/F ratio of the loops with respect to various variables were established.

**Conclusions:**

Increasing the loop height can increase the M/F ratio of the loop. This increasing trend is, especially, much more significant in T-loops compared with vertical loops and L-loops. In vertical loops, increasing the ring radius is much more effective than increasing the loop height to increase the M/F ratio of the loop. Compared with SS, TMA archwire loops can generate a higher M/F ratio due to its lower elastic modulus. Loops with a small cross-sectional area and high activation force can generate a high M/F ratio. The introduction of a leg step to loops does not increase the M/F ratio of loops.

## Background

Orthodontic space closure can be achieved using sliding or loop mechanics. In sliding mechanics, friction is generated between the wire and the bracket, which may decrease the rate of tooth movement [1–3]. In loop mechanics, which is a frictionless technique, forces generated by the loops can be directly transmitted to the teeth without losing force or moment [4–6]. Different loop designs have been introduced, such as vertical [1, 2], T- [3], L-, and teardrop loops, among others. The M/F ratio can control tooth movement patterns [7, 8]; small differences in the M/F ratio result in different tooth movement patterns [9].

Great attention must be paid to the mechanical properties of loops to deliver a predetermined M/F ratio to a tooth. Nowadays, clinical judgment for most orthodontics depends on experience. Clearly, rigorous engineering approaches are needed to study the mechanical properties of loops and identify the dominant design factors that can be used to control loop behavior. Several studies [10–14] examined the ability of a loop design to generate various M/F ratios, especially a sufficiently high M/F ratio for tooth translation and the loop characteristics that determine this ratio.

The mechanical properties generated by loops depend on many confounding factors. The influence of loop material and shape, end conditions (ligation methods), and activation render analysis difficult. These influencing factors can be divided into two groups: one related to loop design and the other related to loop activation. The former includes loop material, cross-sectional dimensions, and configuration geometry, which fundamentally determine the delivered force system of the loop.

Techalertpaisarn and Versluis [15–17] showed that the loop properties varied with loop configuration and position, highlighting that clinicians should understand the specific characteristics of each loop configuration so as to most effectively exploit them for the desired tooth movements. Burstone and Koenig [18] demonstrated that the M/F ratio increased with the loop height and gingival-side width and decreased with the occlusal-side width. However, limited information is available regarding the quantitative mechanical properties of loops. Quantitative knowledge of the mechanical properties of loops allows clinicians to make an informed decision when selecting and designing loops.

Systematic determination of the biomechanical characteristics of loops is not possible clinically. However, these mechanical properties can be evaluated using the finite element method (FEM), which is a computer simulation technique used to simulate and predict the mechanical properties of loops, allowing accurate control of loop shape, properties, fixation, and loading [16, 19, 20].

Considering this, the present study aimed to systematically evaluate the mechanical characteristics, especially the M/F ratio, of the vertical, L- and T-loops using the Abaqus 6.14 software, with a view to ascertaining the quantitative effect of the variables, such as elastic modulus, cross-sectional dimensions, loop configuration geometric dimensions, and activation force, on the generated M/F ratio. A detailed perusal of the changes in mathematical trends suggests that a better method of designing the loops can provide an accurate and constant M/F ratio. The investigated quantitative principles of mechanics can be applied to the orthodontic loop design.

## Methods

### Geometric modeling of the loops

**Fig. 1:**
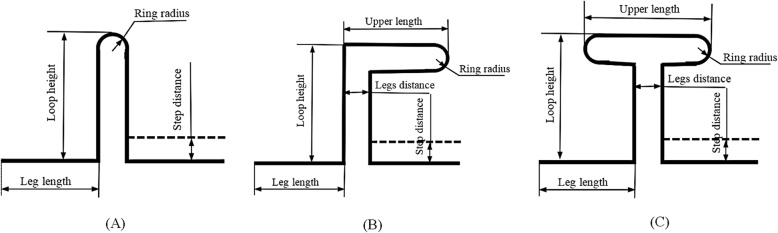
Geometric dimensions of the basic (**a**) vertical loop, (**b**) L-loop, and (**c**) T-loop

Three types of closing loop designs were evaluated in the present study: vertical, L- and T-loops. These three designs and their basic dimensions are shown in Fig. 1a-c.

Six vertical loop variations, 9 L-loop variations, and 9 T-loop variations were evaluated. These variations and their ranges and intervals are listed in Table 1. A total of 141 orthodontic loops were subjected to 3D analysis using FEM. These loop models were all constructed using the Solidworks 2017 software.

**Table 1:** Variations in vertical, L- and T-loops, and their range and intervals

Variations	Vertical loop	L-loop	T-loop
Loop height	3–10 mm at a 1-mm interval	3–10 mm at a 1-mm interval	3–10 mm at a 1-mm interval
Ring radius	0.5–4 mm at a 0.5-mm interval	0.5–4 mm at a 0.5-mm interval	0.5–4 mm at a 0.5-mm interval
Leg length	2–10 mm at a 2-mm interval	2–10 mm at a 2-mm interval	2–10 mm at a 2-mm interval
Leg step distance	0–2 mm at a 0.5-mm interval	0–2 mm at a 0.5-mm interval	0–2 mm at a 0.5-mm interval
Legs distance	–	1–3 mm at a 0.5-mm interval	1–3 mm at a 0.5-mm interval
Upper length	–	4–10 mm at a 2-mm interval	4–10 mm at a 2-mm interval
Elastic modulus	66 GPa [20]	66 GPa [20]	66 GPa [20]
	69 GPa [21]	69 GPa [21]	69 GPa [21]
	90 GPa [22]	90 GPa [22]	90 GPa [22]
	157.6 GPa [17]	157.6 GPa [17]	157.6 GPa [17]
	168 GPa [20]	168 GPa [20]	168 GPa [20]
	200 GPa [23]	200 GPa [23]	200 GPa [23]
Cross-section	0.016″ × 0.022″ [24]	0.016″ × 0.022″ [24]	0.016″ × 0.022″ [24]
	0.017″ × 0.025″ [24]	0.017″ × 0.025″ [24]	0.017″ × 0.025″ [24]
	0.018″ × 0.025″ [24]	0.018″ × 0.025″ [24]	0.018″ × 0.025″ [24]
	0.019″ × 0.025″ [25]	0.019″ × 0.025″ [25]	0.019″ × 0.025″ [25]
Activation force	1–3 N at a 0.5-N interval	1–3 N at a 0.5-N interval	1–3 N at a 0.5-N interval

### Conversion of the geometric model into a 3D finite element model

The solid loop models were imported into the Abaqus 6.14 software, and a 3D finite element model was created. The high-precision eight-node hexahedron element and structural mesh techniques were used to establish the precise finite element loop models. The approximate global element size of the mesh was set as 0.1. Approximately 9000, 13,000, and 13,000 elements were used for the vertical, L-, and T-loops, respectively.

### Material properties

The materials of the loops were assumed to be SS and TMA. The elastic modulus used are listed in Table 1; the Poisson ratio was equal to 0.3.

### Boundary conditions and applied load

**Fig. 2:**
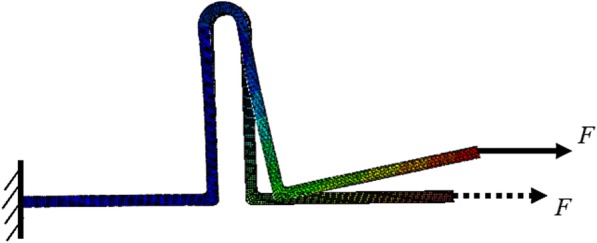
Boundary conditions and the applied load

For modeling activation similar to previous studies [19, 26], one end of the loop was totally fixed in all directions to prevent rigid bodily motion, simulating the ligation of the archwire to the bracket, and a horizontally activated force was applied to the nodes at the other end (Fig. 2).

## Results

### Highest von Mises stress and displacement of the loops

The loop strength should be sufficient, and the highest stress should not reach the yield strength during treatment. Following activation, the loops deformed and the moment was generated at the end of the loop delivered to the tooth. For mechanical knowledge, the generated moment was associated with loop deformation. Therefore, the simulation results of the highest von Mises stress and displacement in the height direction of the loops were recorded.

**Fig. 3:**
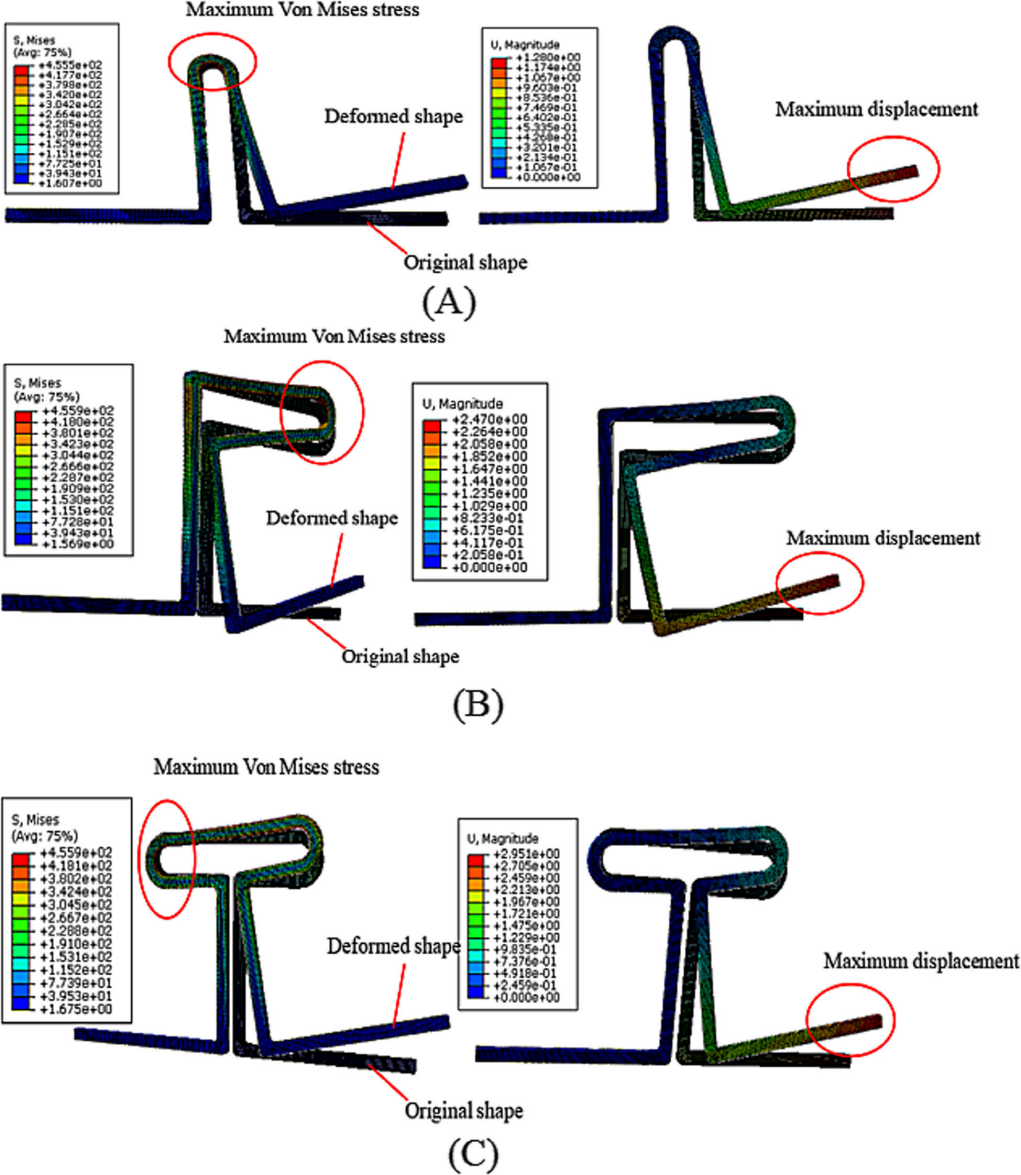
Stress and displacement distribution trends in the basic (**a**) vertical loop, (**b**) L-loop, and (**c**) T-loop

The highest von Mises stress was located on the inner top surface of the ring in the vertical loops (Fig. 3a), on the inner right surface of the ring in the L-loops (Fig. 3b), and on the inner left surface of the ring in the T-loops (Fig. 3c). Also, the highest von Mises stress was always located on the inner surface of the ring. Figure 3 shows that the maximum displacement of the loops was always located on the moving legs.

**Fig. 4:**
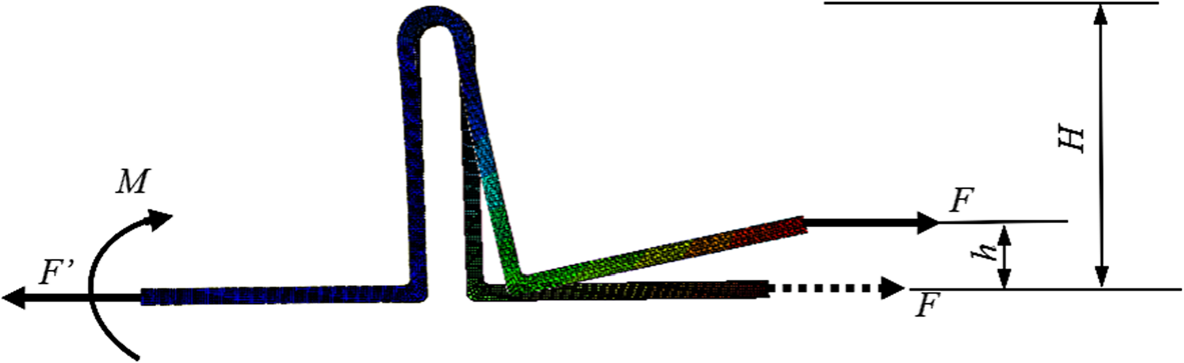
Generated force and moment in the vertical loop following activation

The deformed vertical loop was used to illustrate the calculation of the generated moment of the loop following activation, as shown in Fig. 4. This loop system should satisfy the equilibrium equation; thus, a horizontal force in the opposite direction must exist at the fixed end for *F*^′^ = *F* (Fig. 4). Due to the deformation of the loop, the active lines of the two forces were not collinear; thus, a moment *M* = *F* • *h* acting at the fixed end should exist to maintain the rotational equilibrium (Fig. 4), where *h* is the maximum displacement in the height direction of the moving leg end. For calculating the M/F value, *M*/*F* = (*F* • *h*)/*F* = *h*. Therefore, the M/F ratio of the loop was equal to the displacement in the height direction of the moving leg end.

For the deformation induced by activation, the value of *h* was always smaller than the loop height *H*, which could account for the results obtained by Chacko et al. [20], who stated: “no loop can attain an M/F ratio greater than its height.” Sumi et al. [14] also demonstrated that the M/F ratio generated by the T-loop or the Opus loop could never be higher than its vertical height.

### Mechanical properties of vertical loops

#### M/F ratio

**Fig. 5:**
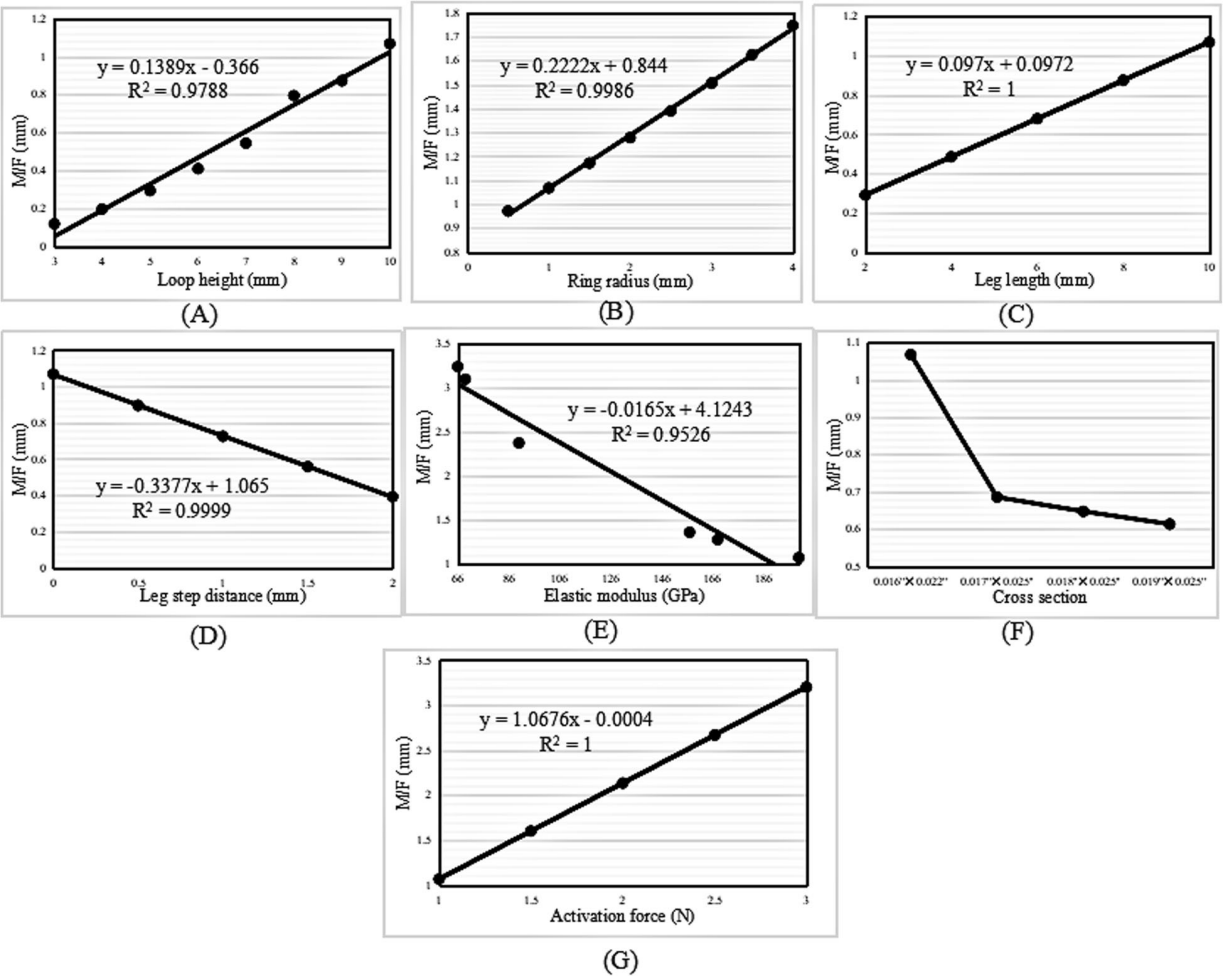
Change trend in the M/F ratio with increasing (**a**) loop height, (**b**) ring radius, (**c**) leg length, (**d**) leg step distance, (**e**) elastic modulus, (**f**) cross-sectional area, and (**g**) activation force in the vertical loop

The M/F value increased linearly with increases in the loop height, ring radius, leg length, and activation force (Fig. 5a-c, g), and decreased linearly with increases in the leg step distance and elastic modulus (Fig. 5d-e). A linear relationship was illustrated by deriving the regression equations. Figure 5f shows that the M/F ratio decreased as the cross-sectional area increased. The maximum M/F ratio was found in the archwire with the lowest cross-sectional area of 0.016″ × 0.022″.

#### von Mises stress

**Fig. 6:**
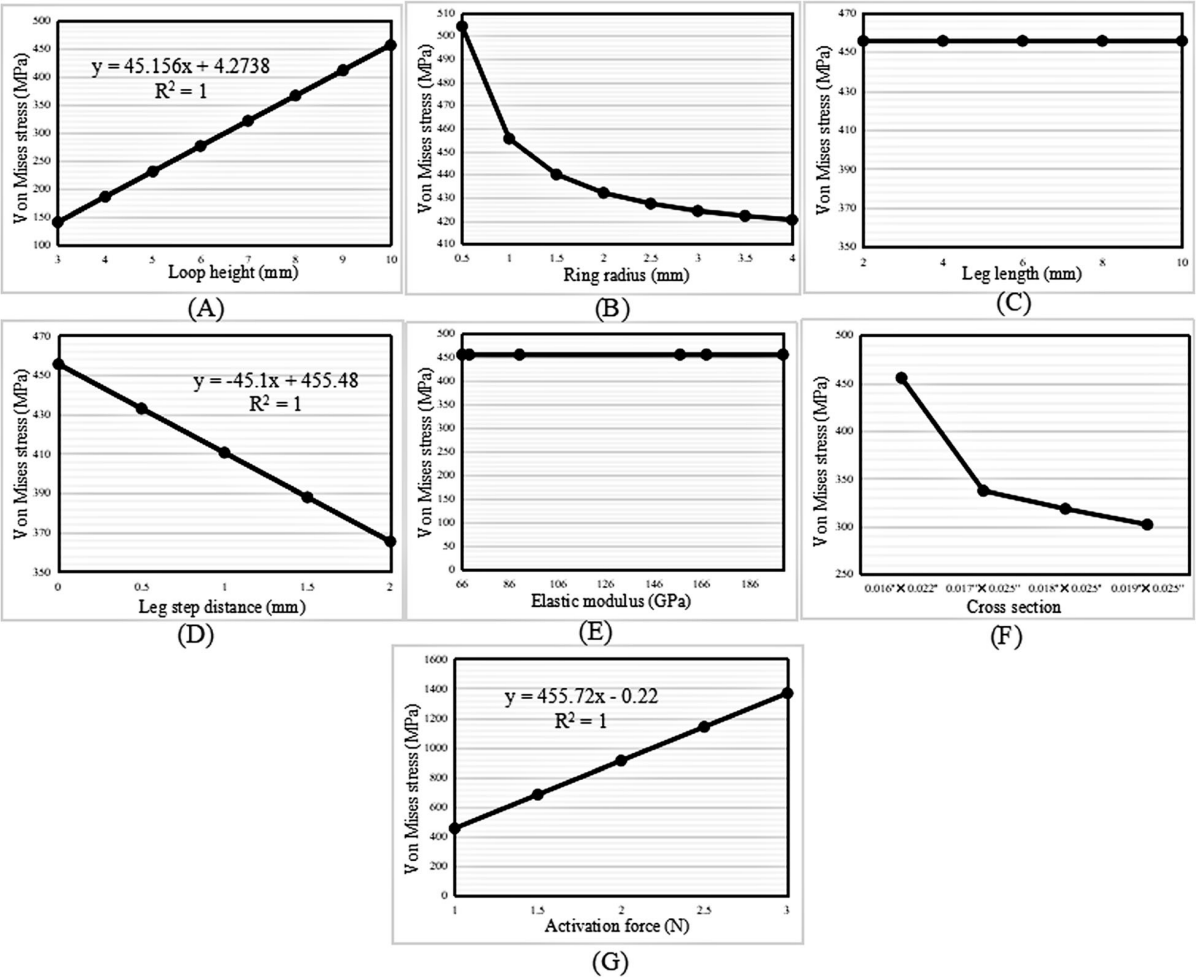
Change trend in the highest von Mises stress with increasing (**a**) loop height, (**b**) ring radius, (**c**) leg length, (**d**) leg step distance, (**e**) elastic modulus, (**f**) cross-sectional area, and (**g**) activation force in the vertical loop

The highest von Mises stress of the loops increased linearly with increases in the loop height and activation force (Fig. 6a and g); however, the highest von Mises stress decreased with increases in the ring radius, leg step distance, and cross-sectional area (Fig. 6b, d, and f). Figure 6c and e shows that the highest von Mises stress of the loops remained constant during the variation in the leg length and elastic modulus.

### Mechanical properties of the L-loops

#### M/F ratio

**Fig. 7:**
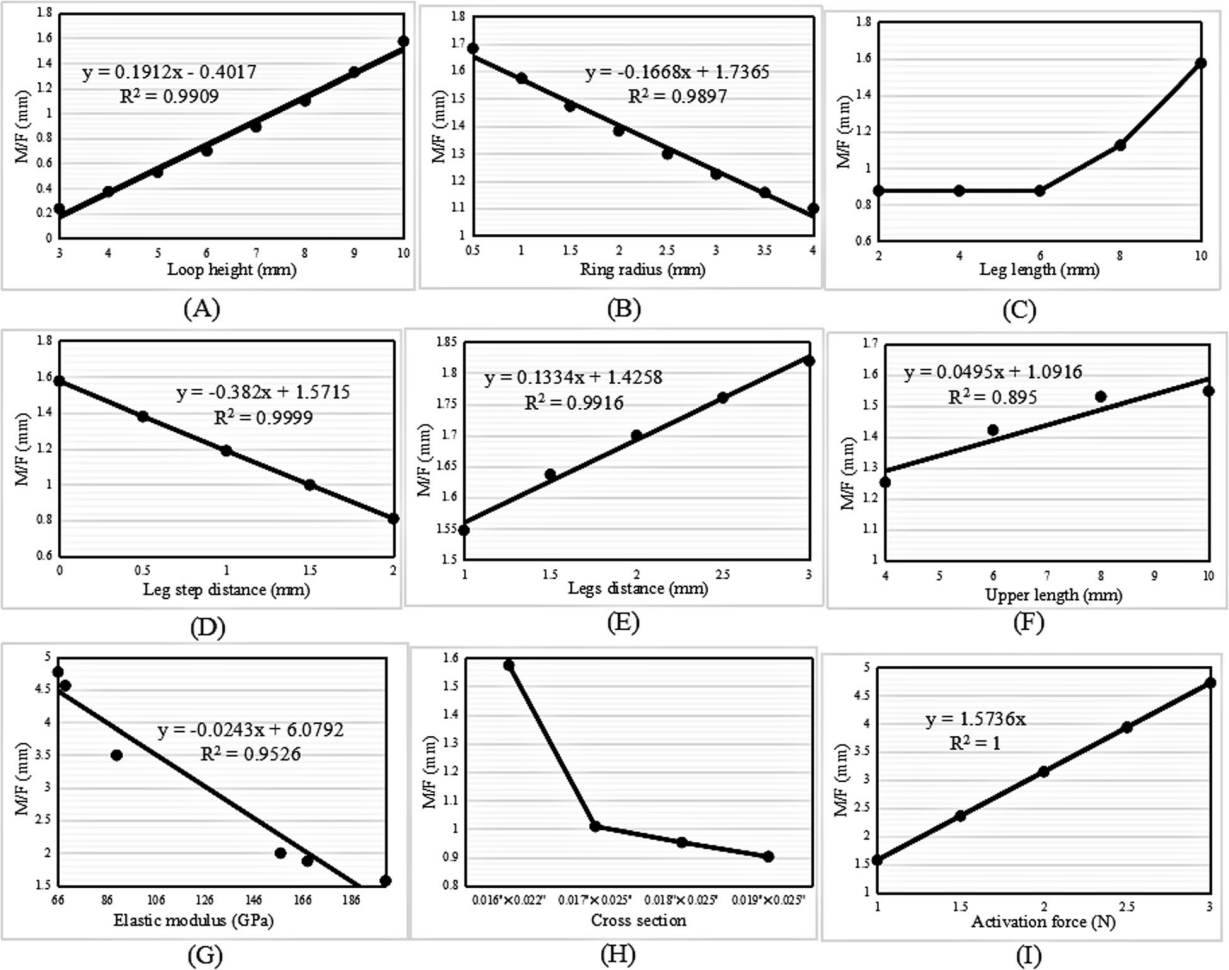
Change trend in the M/F ratio with increasing (**a**) loop height, (**b**) ring radius, (**c**) leg length, (**d**) leg step distance, (**e**) legs distance, (**f**) upper length, (**g**) elastic modulus, (**h**) cross-sectional area, and (**i**) activation force in the L-loop

The M/F ratio increased with increases in the loop height, legs distance, upper length, activation force, and leg length; the former four of these were linear increases, and the latter was almost an exponential increase (Fig. 7a, e, f, i, and c).

The M/F ratio decreased linearly with increases in the ring radius, leg step distance, and elastic modulus (Fig. 7b, d, and g). The change trend in the M/F ratio with increases in the cross-sectional area was the same as that seen in the vertical loops (Fig. 7h).

#### Von Mises stress

**Fig. 8:**
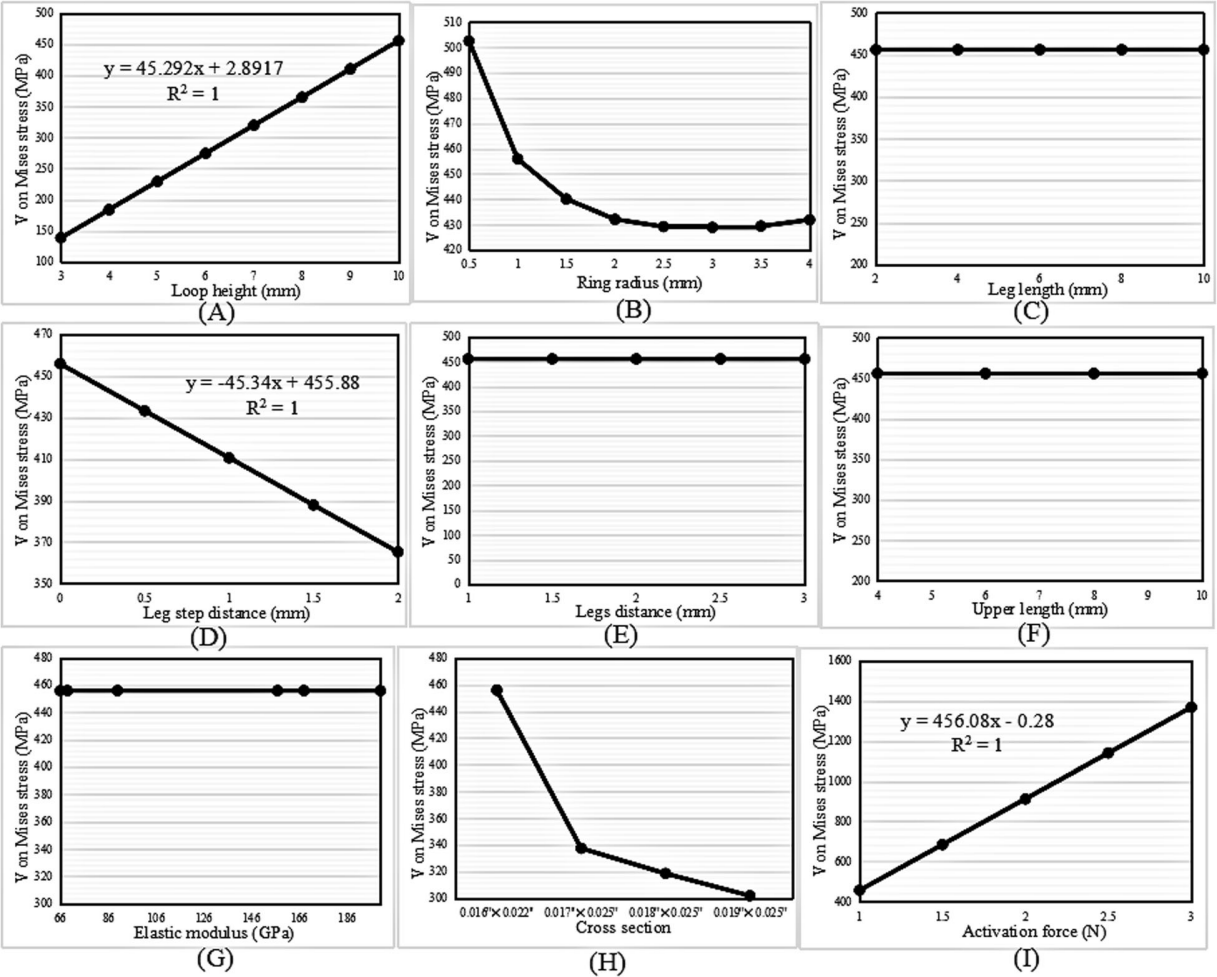
Change trend in the highest von Mises stress with increasing (**a**) loop height, (**b**) ring radius, (**c**) leg length, (**d**) leg step distance, (**e**) legs distance, (**f**) upper length, (**g**) elastic modulus, (**h**) cross-sectional area, and (**i**) activation force in the L-loop

The change trends in the highest von Mises stress with increases in the loop height, activation force, ring radius, leg step distance, cross-sectional area, leg length, and elastic modulus were the same as those in the vertical loops (Fig. 8a-d and g-i). Figure 8e and f shows that the highest von Mises stress of the loops remained the same on variation in the legs distance and upper length.

### Mechanical properties of the T-loops

#### M/F ratio

**Fig. 9:**
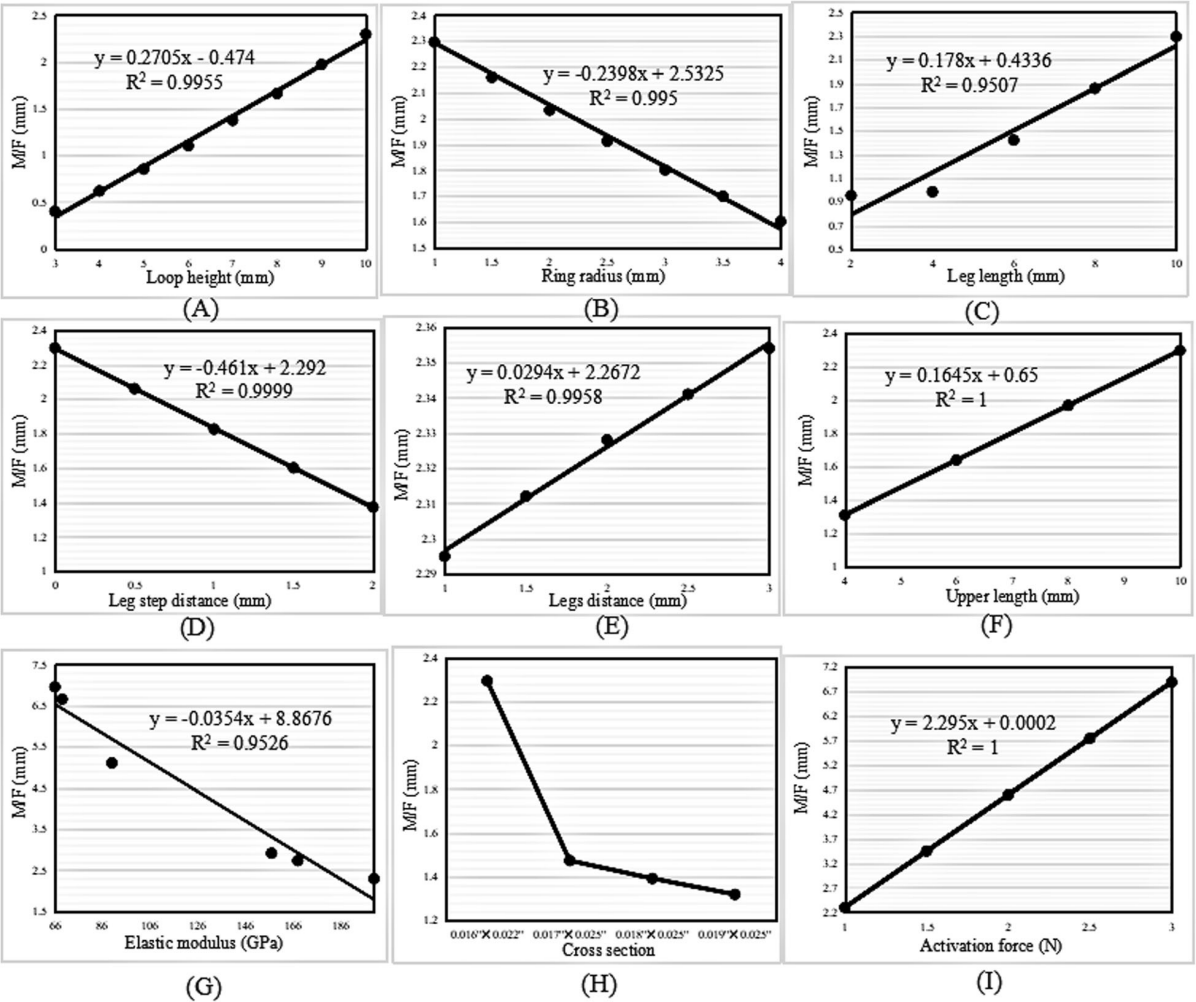
Change trend in the M/F ratio with increasing (**a**) loop height, (**b**) ring radius, (**c**) leg length, (**d**) leg step distance, (**e**) legs distance, (**f**) upper length, (**g**) elastic modulus, (**h**) cross-sectional area, and (**i**) activation force in the T-loop

The M/F ratio increased linearly with increases in the loop height, leg length, legs distance, upper length, and activation force, and decreased linearly with increases in the ring radius, leg step distance, elastic modulus, and cross-sectional area (Fig. 9a-i).

#### Von Mises stress

**Fig. 10:**
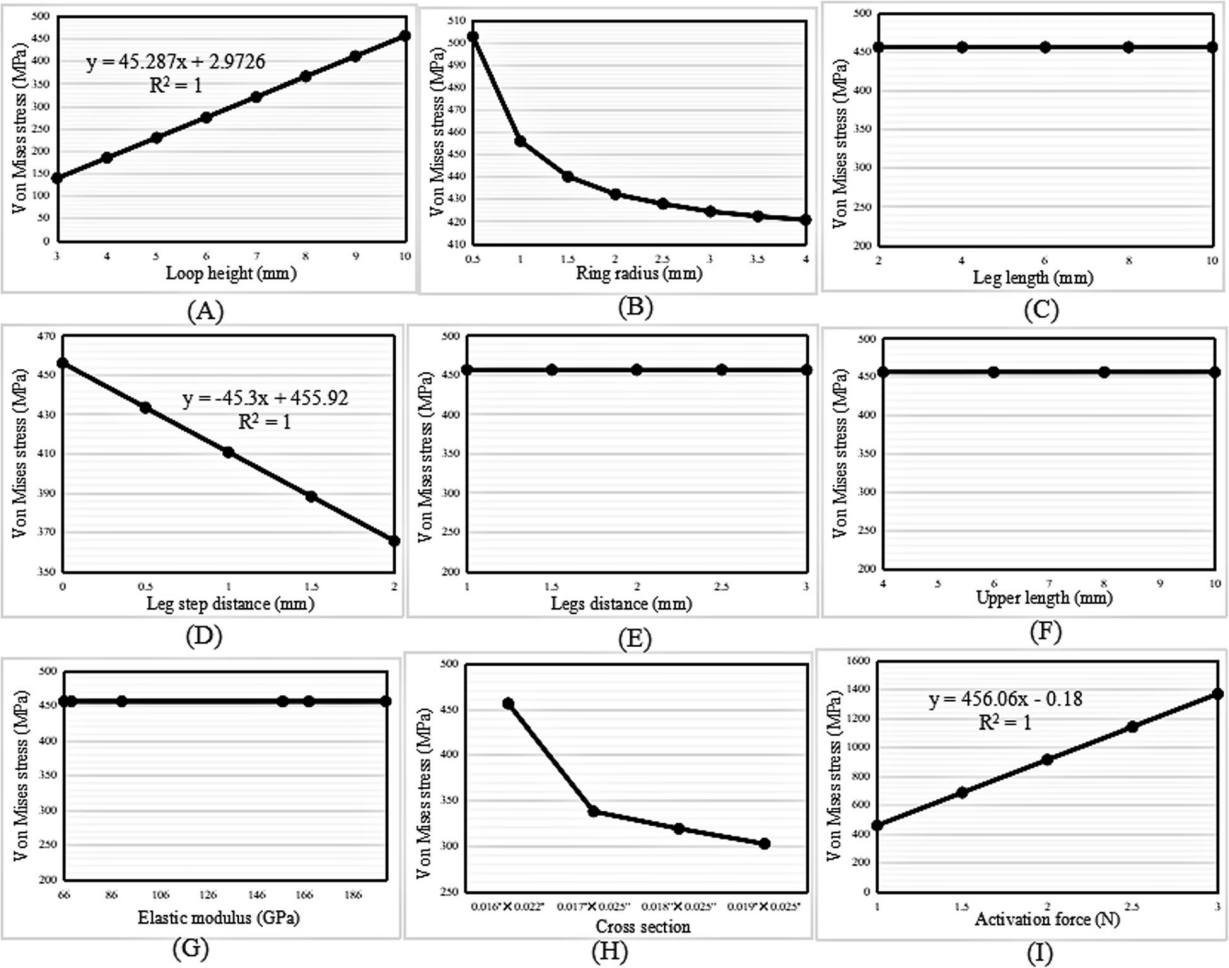
Change trend in the highest von Mises stress with increasing (**a**) loop height, (**b**) ring radius, (**c**) leg length, (**d**) leg step distance, (**e**) legs distance, (**f**) upper length, (**g**) elastic modulus, (**h**) cross-sectional area, and (**i**) activation force in the T-loop

Figure 10 shows that the change trends in the highest von Mises stress with various variables were similar to those in the L-loops.

## Discussion

### Loop height

The M/F ratio and the highest von Mises stress generated by vertical, L- and T-loops increased linearly with increases in the loop height. In other words, the higher the loop height, the larger the M/F ratio generated by the loop. This finding was consistent with those of other studies [13, 18, 26–28]. Ribeiro et al. [13] demonstrated that the M/F ratio increased with increases in the loop height of composite nickel-titanium T-loops. For minor changes in the loop height, the conversion of conventional T-loops into M-loops would increase the M/F ratio. Muñoz-Rendón [27] reported that the convex upper bend of M-loops produced an increased total loop moment compared with T-loops because the upper convex bending increased the total loop height. Moreover, Burstone and Koenig [18] reported that the height of the loop matters more than the length.

The regression equations for the M/F ratio with increases in the loop height of the vertical, L- and T-loops were *y* = 0.1389*x* − 0.366, *y* = 0.1912*x* − 0.4017, and *y* = 0.2705*x* − 0.474, respectively. The slopes of the regression equations indicate an increased rate of the M/F ratio. The slope of the T-loop was the largest, 0.2705; therefore, the increase in the M/F ratio in the T-loop was the highest, followed by the L-loop and the vertical loop.

### Ring radius

The M/F ratio increased linearly with an increase in the ring radius of the vertical loops; however, it decreased linearly in the L- and T-loops. The regression equations were *y* = 0.2222*x* − 0.844, *y* =  − 0.1668*x* + 1.7365, and *y* =  − 0.2398*x* + 2.5325, respectively. Moreover, the highest von Mises stress decreased exponentially with an increase in the ring radius.

The aforementioned results of the vertical loops showed that the regression slope of the ring radius, 0.2222, was higher than that of the loop height, 0.1389. Therefore, it was inferred that increasing the ring radius was much more efficient than increasing the loop height when the goal was to increase the M/F ratio in vertical loops. In particular, unlike increases in the loop height, increases in the ring radius could decrease the stress. Nevertheless, the ring radius should be kept to a minimum to gain a high M/F ratio in L- and T-loops, as long as the stress strength allows.

### Legs distance and upper length

The extra dimensions of the legs distance and upper length in the L- and T-loops had the same effect as that of the ring radius in the vertical loops. The M/F ratio of the L- and T-loops increased linearly with increases in the legs distance and upper length. Moreover, the highest von Mises stress remained constant upon increases in the legs distance and upper length.

The regression equations of the M/F ratio with increases in the legs distance and upper length were *y* = 0.133*x* − 0.14258 and *y* = 0.0495*x* + 1.0916 in the L-loops, and *y* = 0.0294*x* + 2.2672 and *y* = 0.1645*x* + 0.65 in the T-loops, respectively. This indicated that the increase in the M/F ratio with an increase in the legs distance was much higher than that with an increase in the upper length of the L-loops, since 0.133 > 0.0495. However, the increase in the M/F ratio with an increase in the upper length was much higher than that with an increase in the legs distance of the T-loops, since 0.1645 > 0.0294. Despite this, the increase was still lower than that with an increase in the loop height, since 0.1912 > 0.133 and 0.2705 > 0.1645 in the L- and T-loops, respectively. Increasing the legs distance in L-loops and increasing the upper length in T-loops remained the options to increase the M/F ratio, especially since the loop stress was not increased.

### Leg length

The M/F ratio increased with an increase in the leg length in all three types of loops, with the change trends in the vertical and T-loops being linear. The regression equations were *y* = 0.097*x* + 0.0972 and *y* = 0.178*x* + 0.4336, respectively. Moreover, the highest von Mises stress remained constant with an increase in the leg length. In the T-loop, the increase in the M/F ratio (0.178) with an increase in the leg length was higher than that with an increase in the upper length (0.1645), but lower than that with an increase in the loop height (0.2705). Therefore, increasing the leg length was also an option to increase the M/F ratio, especially in T-loops.

### Leg step distance

The M/F ratio decreased linearly with an increase in the leg step distance of vertical, L- and T-loops. Obviously, the introduction of steps in legs decreases the M/F ratio of loops; therefore, leg steps should be avoided when designing vertical, L- and T-loops.

### Elastic modulus

The M/F ratio decreased linearly with an increase in the elastic modulus. Moreover, the elastic modulus had no influence on the highest von Mises stress of the loops. When the material was taken as TMA, with an elastic modulus of 66 GPa, the generated M/F ratio was 3.235 mm, 4.768 mm, and 6.955 mm in the vertical, L- and T-loops, respectively. When the material was taken as SS, with an elastic modulus of 200 GPa, the generated M/F ratio was 1.067 mm, 1.574 mm, and 2.295 mm in vertical, L- and T-loops, respectively. This indicated that the M/F ratio generated by the loops made with TMA was approximately three times larger than that generated by the loops made with SS. These results explained why Safavi et al. [10] found that SS T-loops failed to deliver the optimum M/F ratio of 10:1, but Rose et al. [12] found that the optimal M/F ratio for orthodontic translation could be achieved using TMA T-loops.

Considering the loop configuration in the basic sizes, the M/F ratio generated by the T-loops was the highest, followed by the L-loops, and that generated by the vertical loops was the lowest. In other words, T-loops could generate a higher M/F ratio than vertical or L-loops with similar dimensions.

Burstone and Koenig [18] and Safavi [10] demonstrated that the moment-to-force ratio generated by T-loops was higher than that generated by vertical loops of the same loop height. Moreover, Chacko et al. [20] concluded that a T-loop was preferred over a closed helical loop.

The basic T-loop in the present study, with an elastic modulus of 66 GPa, generated an M/F ratio of 6.955 mm. According to the aforementioned discussion, this M/F ratio could be further increased by increasing the loop height, leg length, legs distance, and upper length. Therefore, it was possible for a T-loop to provide an M/F ratio as high as 10 mm to accomplish tooth translational movement. Choosing an appropriate archwire material that had the lowest elastic modulus and sufficient strength was much more feasible than increasing the geometric dimensions, due to vestibular space limitations.

As shown by Burstone [6], among the normally used loops, T-loops made of beta-titanium alloy were considered one of the best types, since they produced the highest M/F ratio.

### Cross-sectional area

The M/F ratio decreased with an increase in the cross-sectional area. For the four commonly used archwire cross-sectional areas, the minimum area of 0.016″ × 0.022″ generated a higher M/F ratio than that of the other areas in loops of the same type, dimensions, and material parameters. However, the highest von Mises stress of the loops increased with a decrease in the cross-sectional area. Therefore, the archwire cross-sectional area should be as small as possible to gain a high M/F ratio, if the loop strength is sufficient.

Almeida et al. [24] also demonstrated that the M/F ratio was dependent on the wire size. The larger wires were found to produce higher forces, with a slight increase in the moment; however, the M/F ratio produced by the 0.016″ × 0.022″ wire was the highest compared with that produced by 0.017″ × 0.025″ and 0.017″ × 0.025″ wires.

### Activation force

The M/F ratio increased linearly with increases in the activation force of the vertical, L- and T-loops. The magnitude of the activation force is usually determined according to the stress tolerance of periodontal tissues. According to the literature [29, 30], a range of 0.74–1.96 N is needed to obtain satisfactory orthodontic movement.

In summary, the archwire material, cross-sectional area, loop configuration and dimensions, and activation force have a great influence on the generated M/F ratio of the vertical, L- and T-loops. It is important to note that the ligation method, load-deflection rate, and other factors may also affect the final generated M/F ratio of a loop during clinical treatment and should also be considered in future investigations.

## Conclusions

This study was novel in using the FEM combined with static analysis theory to precisely analyze the influence degree and trend of various parameters on the loop force system, especially the M/F ratio.

Increasing the loop height can increase the M/F ratio of the loop. This increasing trend is, especially, much more significant in T-loops compared with vertical loops and L-loops. In vertical loops, increasing the ring radius is much more effective than increasing the loop height to increase the M/F ratio of the loop. Compared with SS, TMA archwire loops can generate a higher M/F ratio due to its lower elastic modulus. Loops with a small cross-sectional area and high activation force can generate a high M/F ratio. The introduction of a leg step to loops does not increase the M/F ratio of loops.

## Data Availability

All data generated or analysed during this study are included in this published article.

## Abbreviations

FEM: Finite element method
M/F ratio: Moment/force ratio
SS: Stainless steel
TMA: Titanium molybdenum alloy
